# Roles of auxin pathways in maize biology

**DOI:** 10.1093/jxb/erad297

**Published:** 2023-07-26

**Authors:** Craig L Cowling, Linkan Dash, Dior R Kelley

**Affiliations:** Department of Genetics, Development, and Cell Biology, Iowa State University, Ames, IA 50011, USA; Department of Genetics, Development, and Cell Biology, Iowa State University, Ames, IA 50011, USA; Department of Genetics, Development, and Cell Biology, Iowa State University, Ames, IA 50011, USA; MPI of Molecular Plant Physiology, Germany

**Keywords:** Auxin, biosynthesis, hormones, maize, metabolism, roots, seeds, shoot architecture, transporters

## Abstract

Phytohormones play a central role in plant development and environmental responses. Auxin is a classical hormone that is required for organ formation, tissue patterning, and defense responses. Auxin pathways have been extensively studied across numerous land plant lineages, including bryophytes and eudicots. In contrast, our understanding of the roles of auxin in maize morphogenesis and immune responses is limited. Here, we review evidence for auxin-mediated processes in maize and describe promising areas for future research in the auxin field. Several recent transcriptomic and genetic studies have demonstrated that auxin is a key influencer of both vegetative and reproductive development in maize (namely roots, leaves, and kernels). Auxin signaling has been implicated in both maize shoot architecture and immune responses through genetic and molecular analyses of the conserved co-repressor RAMOSA ENHANCER LOCUS2. Polar auxin transport is linked to maize drought responses, root growth, shoot formation, and leaf morphogenesis. Notably, maize has been a key system for delineating auxin biosynthetic pathways and offers many opportunities for future investigations on auxin metabolism. In addition, crosstalk between auxin and other phytohormones has been uncovered through gene expression studies and is important for leaf and root development in maize. Collectively these studies point to auxin as a cornerstone for maize biology that could be leveraged for improved crop resilience and yield.

## Teaser

Plant morphogenesis is influenced by the action of phytohormones such as auxin. This review will focus on current research studies that shape our understanding of auxin pathways in maize.

## Introduction

The pervasiveness and longevity of auxin research among plant biologists is well appreciated (and often lamented). Some of the earliest reports on the roles of auxin in maize indicate that this hormone plays a key role in controlling organ size and growth in response to light (Overbeek, 1938; Briggs *et al.*, 1957; Briggs, 1963). The evolution and conservation of auxin pathways in land plants has been recently reviewed (Matthes *et al.*, 2019; Kutschera and Khanna, 2020; Ramos Báez and Nemhauser, 2021; Ang and Østergaard, 2023). In this review, we will focus our discussion towards a synthesis of our current knowledge in the field regarding auxin signaling, biosynthesis, metabolism, and transport specifically in maize based on publications and preprints. Auxin research in maize remains an area of promise and opportunity, with many knowledge gaps remaining and a strong potential to influence crop resilience.

### Nuclear auxin signaling and perception in maize

Auxin signaling has been ascribed to several distinct pathways that have been recently reviewed (Ramos Báez and Nemhauser, 2021; Ang and Østergaard, 2023). The canonical and conserved nuclear auxin signaling pathway in maize occurs via co-receptor pairs consisting of a TRANSPORT INHIBITOR (TIR1)/AUXIN F-BOX (AFB) family member and an INDOLEACETIC ACID-INDUCED PROTEIN (AUX/IAA) (Kepinski and Leyser, 2005; Tan *et al.*, 2007; Calderón Villalobos *et al.*, 2012; Prigge *et al.*, 2020). In maize, there are eight annotated TIR1/AFB family members and 38 Aux/IAAs (Matthes *et al.*, 2019). In contrast to many other model plants, loss-of-function studies on the maize TIR1/AFB family have not yet been reported. The TIR1/AFB family has been associated with auxin-mediated gravitropic response in maize coleoptiles (Nishimura *et al.*, 2009) but this has not been verified using genetic approaches. A yeast-based synthetic approach recapitulated the maize nuclear auxin response with the transcriptional co-repressor RAMOSA ENHANCER LOCUS2 (REL2), ZmAFB2/3, and several ZmIAA proteins (Ramos Báez *et al.*, 2020). In this study, the authors were able to demonstrate that REL2 and ZmIAAs can repress AtARF19, an activator AUXIN RESPONSE FACTOR (ARF) (Ramos Báez *et al.*, 2020). Furthermore, the maize auxin receptor ZmAFB2/3 b1 can rapidly induce the degradation of 16 out of 34 ZmIAAs in the presence of auxin at a faster rate compared with AtAFB2. In addition, lower concentrations of auxin were sufficient to elicit responses, with a complete auxin response circuit (ZmARC^Sc^) expressed in *Saccharomyces cerevisiae* cells. The ZmARC^Sc^ consisted of ZmARF2/3, ZmARF27, and either ZmIAA12, ZmIAA16, or ZmIAA27. This result suggests that the maize nuclear auxin receptor ZmARF2/3 is more sensitive than the orthologous Arabidopsis receptor. Moreover, this work highlights the need for expanded functional characterization of nuclear auxin receptors in maize using genetic and/or transgenic approaches in order to better understand how auxin perception influences maize development.

Auxin signaling in maize has also been examined *in planta* using the DR5- and DII-based reporters (Gallavotti *et al.*, 2008b; Mir *et al.*, 2017). Within the developing inflorescence, the *DR5rev::mRFPer* marker is strongly expressed in spikelet pair meristems, the L1 layer of the bract meristem, and glume tips (Gallavotti *et al.*, 2008b). Conversely, auxin responses are low around developing floral meristems (Mir *et al.*, 2017). During root development, auxin response maxima in developing phloem cells are critical for lateral root formation (Jansen *et al.*, 2012). In addition, high auxin activity was reported for the meristematic zone of primary maize roots and around vasculature associated with lateral root primordia in seedlings exposed to heterogenous phosphate conditions (Wang *et al.*, 2020). High auxin responses have also been associated with maize leaf development based on the DII-VENUS and DR5 reporters (Mir *et al.*, 2017; Robil and McSteen, 2023). In both these studies, it was determined that auxin signaling is high in leaf vasculature. Notably, the DII-VENUS sensor showed dynamic activity during the cell cycle, indicating low auxin signaling during telophase and G_1_. High auxin responses in the adaxial protoderm are associated with tertiary vein formation in maize leaves based on DR5 and DII reporter data, linking auxin signaling to organ patterning in this key crop (Robil and McSteen, 2023).

**Fig. 1.**
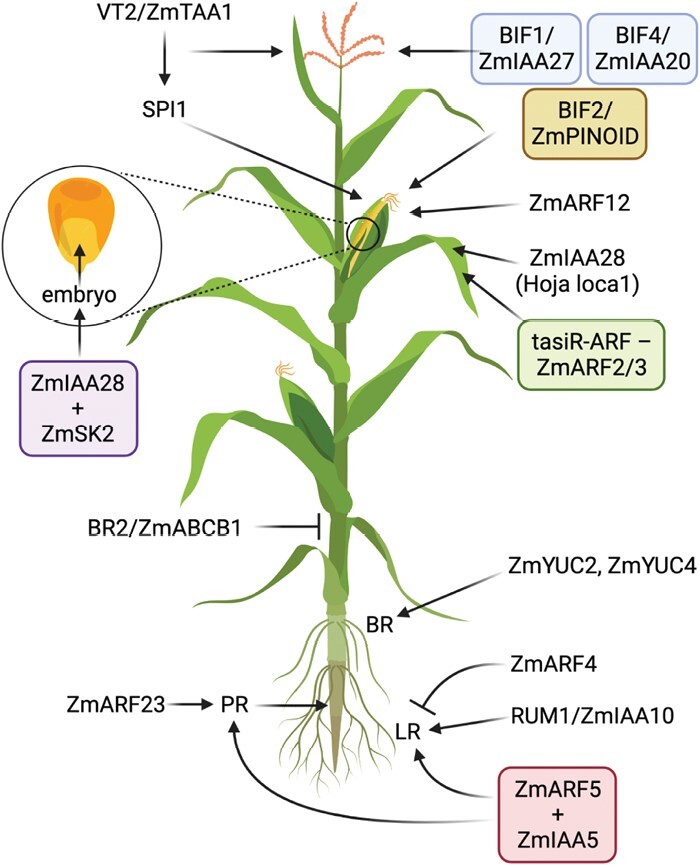
Known auxin pathway genes involved in maize development. Inflorescence development requires auxin biosynthesis (via the VT2/ZmTAA1 and SPI1/YUCCA genes), auxin signaling (through BIF1/ZmIAA27, BIF4/ZmIAA20, and ZmARF12), and auxin transport (BIF2/ZmPINOID). ZmIAA28 (also known as *Hoja loca1*) is implicated in embryogenesis and leaf morphogenesis. The tasi-ARF–ZmARF2/3 module is required for adaxial–abaxial patterning during leaf development. Internode elongation is negatively regulated by auxin transport through BR2, an ABCB family member. Primary root (PR) morphogenesis is positively driven by auxin signaling via RUM1/ZmIAA10, ZmIAA5, ZmARF5, and ZmARF23. ZmARF4 is required to negatively regulate lateral root (LR) formation. Brace root (BR) development is driven by ZmYUC2 and ZmYUC4 activity.

Genetic and functional genomic studies on several maize Aux/IAA proteins have revealed that canonical auxin signaling contributes to maize growth and development (Fig. 1). The dominant *Hoja loca1* (*Oja1*) mutant harbors a lesion in the degron domain of ZmIAA28 which negatively impacts organ initiation and leaf development (Richardson *et al.*, 2020, Preprint). ZmIAA28 has also been implicated in embryogenesis (Wang *et al.*, 2022). The *Barren inflorescence 1* (*bif1*) and *bif4* mutants demonstrate that ZmIAA27/BIF1 and ZmIAA20/BIF4 are both required for maize inflorescence development (Galli *et al.*, 2015). In the absence of *ZmIAA27/BIF1* or *ZmIAA20/BIF4*, lateral organ formation is impaired on inflorescences (both male and female) and polar auxin transport is diminished (Galli *et al.*, 2015). Characterization of the *Rootless with undetectable meristem 1* (*rum1*) mutant has established that ZmIAA10 is important for root morphogenesis and patterning (von Behrens *et al.*, 2011). Loss of *ZmIAA10/RUM1* leads to pleiotropic defects in maize root development, including abnormal vasculature patterning, reduced lateral root formation, and fewer seminal roots (Woll *et al.*, 2005; Zhang *et al.*, 2014). Each of these Aux/IAAs belongs to a separate class and exhibits tissue-specific expression patterns (Walley *et al.*, 2016; Matthes *et al.*, 2019), highlighting the need for additional functional analyses on maize Aux/IAAs to better understand their unique and shared roles in plant development.

**Fig. 2.**
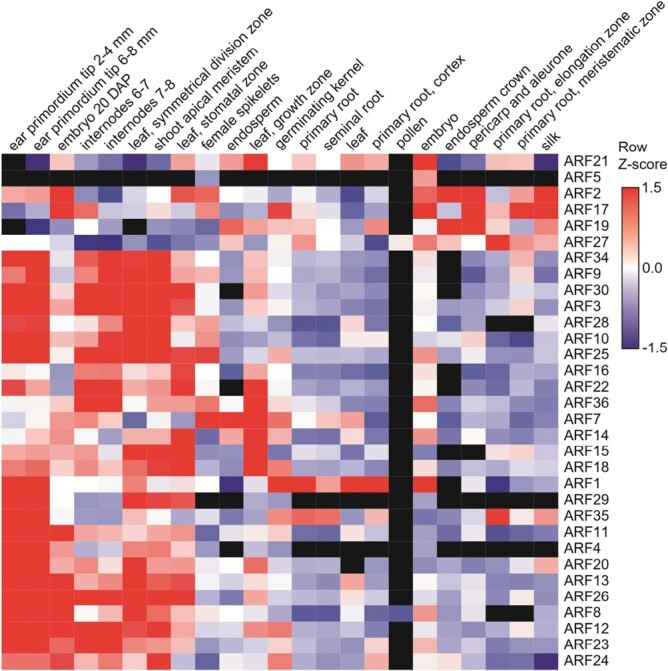
ZmARF expression data during maize development. Transcript abundance for maize ARF genes is from the MaizeGDB. Heirarchical clustering is based on tissue and expression values to identify co-expressed ARF genes.

Auxin signaling also involves the activity of evolutionarily conserved ARF transcription factors (Matthes *et al.*, 2019). ARFs are categorized into three classes, Class A–C, based on their protein domains and associated gene regulation activity (Galli *et al.*, 2018; Mutte *et al.*, 2018). There are 36 annotated ARFs in maize, but the functional roles of these critical gene regulators are not yet well understood (Y. Li *et al*., 2022). In general, *ZmARF* mRNA expression patterns in maize exhibit overlapping patterns with limited tissue specificity (Walley *et al.*, 2016). Hierarchical clustering of *ZmARF* transcriptomic data from Walley *et al.* (2016) demonstrates this finding (Fig. 2). Within the developing inflorescence and axillary meristems, many *ZmARF* transcripts are restricted to specific domains in overlapping patterns (Galli *et al.*, 2015). Nearly all of the maize ARFs are expressed in developing ear tissues, and this may explain why reverse genetics screens to determine ARF function in inflorescences have been hindered to date (Matthes *et al.*, 2019). In leaves, kernels, and reproductive tissues there is also pervasive ARF expression. In contrast, very few ARFs are expressed in root tissues, and only three ARFs (*ZmARF1*, *ZmARF7*, and *ZmARF35*) are expressed in seminal roots (Fig. 2). Several maize ARFs are differentially expressed between two inbreds, suggesting a genetic basis for *ZmARF* expression patterns (Zhang *et al.*, 2017). Numerous maize ARFs have also been associated with drought and salt responses in maize (Wang *et al.*, 2019) and await functional characterization.

Our current knowledge of ARF activity and roles in plant development comes from extensive genetic and biochemical work in other model systems, including Arabidopsis, *Physcomitrium patens*, and *Marchantia polymorpha* (Hardtke *et al.*, 2004; Okushima *et al.*, 2005; Nagpal *et al.*, 2005; Kelley *et al.*, 2012; Lavy *et al.*, 2016; Mutte *et al.*, 2018; Powers *et al.*, 2019; Truskina *et al.*, 2021). To date, only one loss-of-function maize *ARF* study has been published (Wang *et al.*, 2022). A transcriptomic analysis of seed size-associated genes identified *ZmARF12* as a network hub gene that is a negative regulator of kernel length and kernel weight (Wang *et al.*, 2022). *ZmARF12* is in the same clade as *ETTIN/ARF3* (Galli *et al.*, 2015), which does not have known roles in seed size but does impact seed morphology (Kelley *et al.*, 2012). Several gene expression and quantitative genetics studies have implicated numerous *ZmARF* genes in various aspects of maize growth and development (Fig. 1). For example, *ZmARF23* is implicated in embryonic callus formation and primary root development (Y. Li *et al.*, 2022; Liang *et al.*, 2023), while ZmARF4 and ZmARF5 (together with ZmIAA5) are linked to lateral root formation (J. Li *et al*., 2022; Yang *et al.*, 2022). *ZmARF2* and *ZmARF4* have been implicated in phosphate uptake (Sheng *et al.*, 2020) and lateral root formation (J. Li *et al*., 2022), respectively, but have yet to be validated using genetic approaches. *ZmARF4* expression is also positively regulated by nitrate and 1-naphthaleneacetic acid (NAA) treatment (Ravazzolo *et al.*, 2021). Finally, a tasiR–ARF–ZmARF2/3 module has been implicated in establishment of leaf polarity and organogenesis (Dotto *et al.*, 2014). Altogether the genetic studies on Aux/IAA and ARF genes in maize are examples of how phylogenetic relationships may not always track with gene function.

Prior to fertilization, maize ovules exhibit high levels of *DR5:RFP* in the antipodal cells, which may generate an auxin gradient within the embryo sac (Chettoor and Evans, 2015). After fertilization, auxin signaling is implicated in aleurone layer formation during maize kernel development via the activity of two zinc finger transcription factors, NKD1 and NKD2 (Wu and Becraft, 2021). In the absence of *NKD1* and *NKD2*, several *ZmARF* genes show increased mRNA levels and, correspondingly, the *DR5:RFP* reporter shows increased activity in the aleurone layer of *nkd1,2* kernels. A network analysis of NKD targets identified *ZmARF29*, *ZmARF34*, and *ZmARF35* as being repressed by these transcription factors during normal kernel development. During maize embryogenesis, the *DR5:RFP* reporter exhibits complex dynamics in the endosperm and embryo proper, which is consistent with what has been described in Arabidopsis (Chen *et al.*, 2014). Altogether these studies highlight the potential tissue-specific roles among maize ARFs during reproduction and embryogenesis.

A recent analysis of auxin-responsive transcription in maize primary roots used an unsupervised network reconstruction approach to predict gene targets of maize *ZmARF* genes (McReynolds *et al.*, 2022), but these data have yet to be tested *in vivo*. In addition, thousands of *ZmARF* target genes have been identified using large-scale DNA affinity purification followed by sequencing (DAP-seq) in maize (Galli *et al.*, 2018). These two studies indicate that *ZmARF*-binding sites may exhibit sequence variability, and target genes may be both shared and unique. Additional investigation on *in vivo* targets of ZmARF proteins will help elucidate the extent of target overlap among maize ARFs.

### Auxin transport in maize

Polar auxin transport is well known to influence a myriad of plant processes (Carrillo-Carrasco *et al.*, 2023) and is accomplished by several evolutionarily conserved proteins that are also present in monocots (Balzan *et al.*, 2014). The four main families of auxin transporters will be discussed here: AUXIN1/LIKE-AUX1s (AUX/LAX), PIN-FORMED (PIN), PIN-LIKES (PILS), and ATP-binding cassette family B (ABCB). In Arabidopsis, these proteins are localized to the plasma membrane or endoplasmic reticulum and can participate in auxin efflux or influx across membranes. In maize, many of these protein families are greatly expanded and only a few family members have been studied to date using genetic approaches (Matthes *et al.*, 2019). Expression profiling of all annotated *ZmPIN*, *ZmPILS*, *ZmLAX*, and *ZmABCB* genes demonstrated that most auxin transporter family members are induced in shoots but reduced in roots in response to abiotic stresses (drought, salt, and cold) (Yue *et al.*, 2015). This study lays the foundation for future investigation into the roles of auxin transporters in mediating abiotic stresses, which is of key relevance for crops such as maize.

### Maize auxin influx carriers

The AUX/LAX family of proteins are auxin influx carriers that regulate the flow of auxin into the cell (Péret *et al.*, 2012). ZmAUX1 shows tissue-specific root expression patterns (Hochholdinger *et al.*, 2000) but has been linked to shoot development via recent functional analyses. Loss of *ZmAUX1* leads to a reduction in tassel branch number and fewer spikelets per row in both the tassel and ear (Huang *et al.*, 2017; Zhu *et al.*, 2022). *ZmAUX1* and *AtAUX1* share a conserved role in regulating root gravitropism (Marchant *et al.*, 1999; Huang *et al.*, 2017). *In situ* hybridization demonstrates that *ZmAUX1* is localized to the endodermal and pericycle cell layers of the primary root (Hochholdinger *et al.*, 2000). The phenotype of *ZmAUX1* loss of function is severely enhanced in the absence of several auxin pathway genes, such as the *bif2*, *bif4*, and *vanishing tassel 2* (*vt2*) mutants. These double mutants had synergistic phenotypes and displayed pleiotropic growth defects in the plant architecture and lateral organ formation, supporting the idea that auxin pathways work in concert during maize development (Zhu *et al.*, 2022).

### Auxin efflux carriers

The PIN-FORMED (PIN) family of auxin transporters are integral membrane proteins that typically localize to the plasma membrane and the endoplasmic reticulum. PINs are important for directional cell to cell transport and maintaining intracellular homeostasis of auxin for development. In maize coleoptiles, polar transport of indole-3-acetic acid (IAA) is regulated by ZmPIN proteins (Nishimura *et al.*, 2012). Treatment of maize coleoptile tips with an IAA transport inhibitor, 1-naphthylphthalamic acid (NPA), resulted in impaired IAA movement from the tip and suppressed gravitropic bending (Nishimura *et al.*, 2009). Expression patterns of *ZmPIN* family members have been examined extensively using transcriptomics and immunostaining, but very few maize PIN genes have been studied functionally.

In maize there are four genes, *ZmPIN1a*, *ZmPIN1b*, *ZmPIN1c*, and *ZmPIN1d*, that are homologous to Arabidopsis PIN1 (Balzan *et al.*, 2014; Matthes *et al.*, 2019). Note that the current annotations at MaizeGDB for *ZmPIN* genes have switched to *pin1–4* (B73 RefGen_v3), but the earlier PIN1a–d annotations are well established in the literature and thus will be discussed accordingly. *ZmPIN1d*, also known as *Sister-of-PIN1* (*SoPIN1*) (O’Connor *et al.*, 2014), is present in all angiosperms except for *Brassicaceae* (Matthes *et al.*, 2019; Kellogg, 2022). SoPIN1 expression is associated with organogenesis in maize, along with ZmPIN1a and ZmPIN1b, but its functional role awaits further characterization (O’Connor *et al.*, 2014). Both ZmPIN1a and its co-ortholog Arabidopsis PIN1 (AtPIN1) are expressed in developing veins (Gälweiler *et al.*, 1998; Carraro *et al.*, 2006; Scarpella *et al.*, 2006; Gallavotti *et al.*, 2008b; Robil and McSteen, 2023), suggesting a conserved vasculature association between these PIN1 proteins. Furthermore, expression of *ZmPIN1a* and *AtPIN1* within procambial cells appears to occur irrespective of vein patterning, as monocots and eudicots display different venation patterns (Robil and McSteen, 2023).

In tissue-specific expression analyses of the PIN family, *ZmPIN1d* had lower expression in the kernel and during early stages of development, whereas *ZmPIN1a*, *ZmPIN1b*, and *ZmPIN1c* are highly expressed (Forestan *et al.*, 2012; Walley *et al.*, 2016; Li *et al.*, 2019). The ZmPIN1 proteins are up-regulated after fertilization and are important for cellular differentiation during embryogenesis (Forestan and Varotto, 2010) and later development of the inflorescence (Carraro *et al.*, 2006) and root (Li *et al.*, 2019). During embryogenesis, ZmPIN1 shifts within the cell from a radial to a bilateral distribution which differentiates the scutellum and the shoot apical meristem (SAM). Within the SAM, ZmPIN1 proteins remain localized to the primordium initiation site for leaf formation and differentiation of vasculature tissue (Carraro *et al.*, 2006; Gallavotti *et al.*, 2008b; Robil and McSteen, 2023). PIN1 expression has also been associated with the pre-ligule band (PLB) during leaf development, suggesting that it may underpin PLB differentiation (Moon *et al.*, 2013; Johnston *et al.*, 2015; Conklin *et al.*, 2019). However, a recent study indicates that PIN1 expression dynamics in the PLB do not track with differential auxin response at the transcriptional level (Neher *et al.*, 2023, Preprint). Thus, additional investigations into the role of PIN proteins and auxin during ligule formation will be required to better understand this process.

In root development, *ZmPIN1a* and *ZmPIN1b* have different developmental roles from one another. Overexpression of *ZmPIN1a* led to an increase in the number of lateral roots and inhibited their elongation, whereas overexpression of *ZmPIN1b* promoted an increased growth of lateral roots and seminal roots (Li *et al.*, 2019). This work demonstrates the evolutionary neo- and subfunctionalization that can occur among related gene family members. Sister-of-PIN1 (ZmPIN1d/ZmSoPIN1) is more closely related to ZmPIN2 rather than ZmPIN1 (O’Connor *et al.*, 2014). Further studies will be required to determine SoPIN1 expression during maize root development and to identify the extent of functional overlap between ZmPIN1 family members during root morphogenesis.

Three of the four ZmPIN1 proteins are phosphorylated in maize across developmental stages (Walley *et al.*, 2016). In Arabidopsis, the PINOID (PID) protein is a Ser/Thr kinase that directly phosphorylates AtPIN1 and regulates its subcellularization (Friml *et al.*, 2004). The maize ortholog of PID is *BARREN INFLORESCENCE2* (*bif2*). BIF2 can phosphorylate ZmPIN1a *in vitro* and plays a key role in shoot development (McSteen *et al.*, 2007; Skirpan *et al.*, 2009). This suggests that ZmBIF2 and PID may have a conserved role in auxin transport across species. Future investigations into the kinases involved in maize PIN phosphorylation and the functional significance of these post-translational modifications will be of interest to the field to better understand the regulation of auxin efflux carriers.

Phylogenetic analyses of auxin transporters in maize, rice, and Arabidopsis have identified proteins that were previously classified as PINs but are more closely related to the PIN-likes (PILS) proteins in Arabidopsis. Two of these PILS proteins are ZmPINX and ZmPINY/ZmPILS4/ZmPIN14 (Forestan *et al.*, 2012; Balzan *et al.*, 2014; Yue *et al.*, 2015). Notably, the nomenclature among PIN and PILS family members has oscillated between alphabetical, numerical, and alphanumerical, which makes annotation a challenge among this family of auxin transporters. *ZmPILS4* (also called *ZmPINX* and currently annotated as *ZmPIN14*, the ortholog of *AtPILS6*) was identified from a quantitative trait locus (QTL) analysis of root traits in maize (Chen *et al.*, 2022). Extrapolating from lower expression patterns of ZmPILS4 in near isogenic lines, it is predicted that ZmPILS4 has a role in reducing auxin accumulation for nuclear signaling and reduces the differentiation of xylem cells in the root tip and phloem (Chen *et al.*, 2022).

In plants, the ATP-binding cassette subfamily B (ABCB) proteins are auxin transporters that are homologous to the mammalian multi-drug resistance/P-glycoprotein (MDR/PGP) proteins. The dwarf mutant *brachytic2* (*br2*) is a loss-of-function mutant for *ZmABCB1/ZmPGP1* (Knöller *et al.*, 2010; Wei *et al.*, 2018; Zhao *et al.*, 2023). The defects in auxin transport of *br2* plants causes a reduction in cell elongation and an overall shortening of the lower internodes (leaf 3 through 7) and an increase of node vasculature (Knöller *et al.*, 2010; Zhang *et al.*, 2019). Tracking of radiolabeled IAA in the *br2* mutant shows a reduction of auxin transport from the meristem to the elongation zone which causes agravitropic growth (McLamore *et al.*, 2010). ZmABCB1/ZmPGP1 also has an important role in auxin efflux in response to aluminum stress. A ZmABCB1/ZmPGP1 mutant has higher levels of auxin accumulation in the roots compared with the control which is depleted of auxin when subjected to aluminum stress (Zhang *et al.*, 2018).

An expression analysis of *ZmABCB* genes shows that most family members are responsive to abiotic stresses. In addition, *ZmABCB15* was induced in response to biotic stress. Overexpression of *ZmABCB15* in a susceptible variety enhanced resistance to maize rough dwarf disease by repressing the replication of rice black-streaked dwarf virus (Yue *et al.*, 2022). Additional genetic studies will need to be carried out to better understand the contributions of ABCB proteins in maize.

### Auxin biosynthesis and metabolism in maize

The biosynthesis of IAA in maize occurs via the indole-3-pyruvic acid (IPA) pathway, whereby tryptophan is converted into IPA by TRYPTOPHAN AMINOTRANSFERASE OF ARABIDOPSIS1 (TAA1/TAR) aminotransferases, and IPA is subsequently converted into IAA by YUCCA flavin mono-oxygenases (Gomes and Scortecci, 2021). Notably, the controversial tryptophan-independent pathway identified via the *orange pericarp* maize mutant is still not well understood (Wright *et al.*, 1991; Nonhebel, 2015; Casanova-Sáez *et al.*, 2021). However, auxin biosynthesis research in maize has played a critical role on delineating the IPA route to produce IAA *in vivo*. Positional cloning and functional characterization of the *vanishing tassel2* (*vt2*) mutant led to the discovery that *vt2* is an ortholog of TAA1 (Phillips *et al.*, 2011). Furthermore, genetic analysis of *vt2* and *sparse inflorescence1* (*spi1*), which encodes a YUCCA family member, determined that *vt2* and *spi1* function in the same pathway to produce IAA in maize (Phillips *et al.*, 2011). This same pathway was also confirmed in Arabidopsis using biochemical approaches (Mashiguchi *et al.*, 2011), leading to its widespread acceptance in the field. Auxin biosynthesis across different stages of maize development has been described based on integrated transcriptomic and metabolomic data (Jiang *et al.*, 2022).

There are 14 *YUCCA* genes in the maize genome, but only four have been characterized to date (Gallavotti *et al.*, 2008a; Bernardi *et al.*, 2012; Li *et al.*, 2015; Matthes *et al.*, 2019). In addition to *spi1*, *defective endosperm18* (*de18*) is required for auxin biosynthesis and corresponds to *ZmYUC1* (Bernardi *et al.*, 2012). IAA leves are diminished in *de18* endosperm, and the mutants have smaller kernels (Bernardi *et al.*, 2019). In addition, *de18* kernels contain reduced starch content, suggesting that auxin levels may modulate central metabolism within developing seeds (Bernardi *et al.*, 2019). This finding is consistent with other studies from pea, barley, and Arabidopsis that all suggest that auxin can promote starch biosynthesis (McAdam *et al.*, 2017; Zhang *et al.*, 2019; Amanda *et al.*, 2022) Recent research has shown that two maize *YUCCA* genes, *ZmYUC2* and *ZmYUC4*, are required for proper gravity response in maize brace roots (Zheng *et al.*, 2023). In the *Zmyuc2 Zmyuc4* double mutant, brace root angles are enlarged and the plants exhibit enhanced root lodging resistance (Zheng *et al.*, 2023). Notably, ZmYUC2 was shown to be localized to the cytoplasm while ZmYUC4 was within the endoplasmic reticulum (Zheng *et al.*, 2023). The observed endoplasmic reticulum localization is consistent with an earlier report on the subcellular localization of maize YUCCA proteins, which was proposed to contribute to spatial intracellular auxin patterns (Kriechbaumer *et al.*, 2015). Altogether these studies suggest unique and overlapping functions among maize YUCCA proteins. Continued research on these auxin biosynthetic enzymes will be needed to map out IAA production during maize development and in response to environmental cues.

Auxin metabolism is complex and involves large families of evolutionarily conserved enzymes (Casanova-Sáez *et al.*, 2021). Auxin metabolism can include several distinct biochemical events, such as conversion between auxinic compounds (i.e. indole-3-butyric acid to IAA), conjugation of IAA to sugars and amino acids via the action of GRETCHEN HAGEN3 (GH3) enzymes, and oxidation of IAA (Casanova-Sáez *et al.*, 2021). Recently, it was demonstrated that IAA conjugation and oxidation can occur via a single pathway, termed the GH3–ILR1–DAO pathway, which is critical for auxin inactivation (Hayashi *et al.*, 2021). It is not known yet if this GH3–ILR1–DAO pathway also operates in maize. Maize kernels contain hydrolases that can release free IAA from IAA-glucose (IAA-Glc), presumably to regulate the release of free auxin from storage forms such as IAA-Glc (Jakubowska and Kowalczyk, 2005). Continued efforts to characterize IAA levels, as well as precursors and conjugates, will be needed to better understand how auxin metabolism contributes to maize biology.

### Crosstalk between auxin and other phytohormones in maize

Hormone crosstalk in plants occurs when hormone pathways exhibit synergistic or antagonistic interactions (Liu *et al.*, 2014). Several examples of auxin crosstalk with other phytohormones in plants demonstrate that hormone pathways are tightly connected in maize. Some of these connections are reminiscent of phenomena described in Arabidopsis, but many are unique to maize. For example, auxin and brassinosteroid (BR) crosstalk occurs via ZmIAA28 and ZmSK2 (a BIN2 GSK3-like kinase ortholog) (Wang *et al.*, 2022). BIN2 has also been implicated in Arabidopsis ARF phosphorylation (Vert *et al.*, 2008). Thus, auxin and BR pathways may be integrated via protein phosphorylation events that impact hormone signaling.

Auxin and cytokinins (CKs) have long gone hand-in-hand as they are widely used in tissue culture and propagation. In maize, auxin/CK ratios are critical for callus differentiation (Linsmaier-Bednar and Bednar, 1972; Kang *et al.*, 2022). At the molecular genetic level, several examples of auxin and CK crosstalk have been reported for maize. Characterization of the *aberrant phyllotaxy 1* (*abph1*) mutant has demonstrated that reductions in both auxin and CK are required for proper leaf primordia initiation (Lee *et al.*, 2009). Within maize roots, spatial restriction of auxin and CK between the vasculature and cortical parenchyma, respectively, may contribute to lateral root formation within the pericycle and endodermis (Saleem *et al.*, 2009). Indeed, the relative balance between auxin and CK has been recently shown to control primary root elongation and lateral root formation in maize (Rivas *et al.*, 2022). Notably, a phenotypic analysis of maize seedlings treated with auxin and CK indicates that these two hormones elicit distinct, but overlapping developmental effects on root morphogenesis (Draves *et al.*, 2022). Both auxin and CK have been recently shown to positively influence internode elongation in maize, which may indicate a cooperative mode of action between these two hormones to regulate cell cycle and cell wall dynamics (Ren *et al.*, 2023). In addition, the collective flux of both auxin and CK at the base of a developing maize leaf may help induce cell division and induce gibberellic acid (GA) production (De Vos *et al.*, 2020). Imaging of auxin and CK reporters during embryo sac development indicates that both hormones are active in antipodal cells and the integuments, suggesting overlapping function during ovule formation (Chettoor and Evans, 2015).

Emerging connections between auxin and ethylene have also been reported for maize development. Both ethylene and auxin synthesis are required within maize silks to ensure proper embryo sac differentiation following pollination (Mól *et al.*, 2004). Within the root, auxin and ethylene work in concert to regulate root cap morphogenesis (Ponce *et al.*, 2005). It is worth noting that both maize YUCCA proteins and all five ethylene receptors are localized in the endoplasmic reticulum (Kriechbaumer *et al.*, 2015), suggesting opportunities for crosstalk at the subcellular level. Additional efforts to better understand the relationship between auxin and ethylene during vegetative and reproductive development will be needed in the field, as extensive auxin–ethylene crosstalk has been described in Arabidopsis (Zemlyanskaya *et al.*, 2018; Xu *et al.*, 2020; Wei *et al.*, 2021).

## Conclusion

Auxin is central to growth-related gene function networks across plant species, including maize (Matthes *et al.*, 2019). In order to further elucidate specific roles of auxin pathway genes in maize, additional genetic and molecular approaches will be needed. Because many auxin pathway genes belong to large gene families in maize which exhibit overlapping expression patterns (Matthes *et al.*, 2019), it may be important to consider multiple criteria when performing reverse genetic screens. For example, a focus on protein abundance data rather than transcript levels among genes of interest could help inform which family member(s) may contribute to particular aspects of maize growth and development (Walley *et al.*, 2016). The deployment of stacked guide RNAs to target multiple gene family members simultaneously may also prove to be useful to link genes to function across auxin pathways (Impens *et al.*, 2023). For example, co-expressed ARFs could be simultaneously targeted and subsequently characterized using phenotypic and molecular approaches. Forward genetic screens and quantitative approaches may also be fruitful in linking individual auxin pathway genes to maize morphogenesis.

Another outstanding question in the field is what roles protein phosphorylation play among ARF, Aux/IAA, PIN, and PILS proteins. All of these proteins have been detected to be phosphorylated across maize tissues with spatial specificity (Walley *et al.*, 2016). However it is not currently known how such protein modifications impact the activity of such proteins or which kinase(s) regulate these phosphorylation events. Rapid deployment of transient-based kinase assays (Montes *et al.*, 2022) and/or base editing approaches (Basu and Parida, 2023) to modify phosphorylated amino acids *in vivo* may help shed light on these knowledge gaps.
